# Left Ventricular Assist Device in Advanced Refractory Heart Failure: A Comprehensive Review of Patient Selection, Surgical Approaches, Complications and Future Perspectives

**DOI:** 10.3390/diagnostics14222480

**Published:** 2024-11-07

**Authors:** Antonio Al Hazzouri, Philippe Attieh, Christopher Sleiman, Righab Hamdan, Hilda E. Ghadieh, Bernard Harbieh

**Affiliations:** 1 Department of Biomedical Sciences, Faculty of Medicine and Medical Sciences, University of Balamand, Al-Koura, Tripoli P.O. Box 100, Lebanon; antonio.hazzouri@std.balamand.edu.lb (A.A.H.); philippe.attieh@std.balamand.edu.lb (P.A.); christopherj.sleiman@std.balamand.edu.lb (C.S.); hilda.ghadieh@balamand.edu.lb (H.E.G.); 2 Department of Internal Medicine-Cardiology, Lebanese American Medical Center—Rizk Hospital, Beirut P.O. Box 11-3288, Lebanon; hamdanrighab@gmail.com

**Keywords:** advanced heart failure (HF), biomedical advancements, clinical outcomes, left ventricular assist device (LVAD) therapy, complications, quality of life, survival rates, technological innovations, medical ethics

## Abstract

The management of advanced heart failure (HF) has long posed significant challenges due to its complex and chronic nature. Heart transplantation, while effective, is not always feasible due to the limited availability of donor organs. In this context, long term mechanical circulatory support and mainly left ventricular assist devices (LVADs) have emerged as a vital intervention to fill this gap. LVAD superiority compared to medical therapy for some patients in advanced heart failure has been demonstrated either as a bridge to transplantation or as destination therapy. This literature review provides a comprehensive overview of the effectiveness, challenges, and advancements in the use of LVADs for treating advanced heart failure. It evaluates clinical outcomes associated with LVAD therapy, focusing on survival rates and quality of life improvements. The review synthesizes findings from recent studies, highlighting both the benefits and complications of LVAD implantation, such as infectious risk, thromboembolic events, hemorrhage and device malfunction. Additionally, it explores the latest technological and biomedical advancements in LVAD design, including innovations in biocompatibility, miniaturization, and power management. By examining current research, this review aims to elucidate how LVADs are transforming heart failure treatment and to offer insights into future directions for clinical practice and research.

## 1. Introduction

Heart failure (HF) is defined as a complex clinical syndrome resulting from both structural and functional impairment of blood ejection and ventricular filling [1]. It can be classified into two categories according to the ejection fraction. HF with an ejection fraction ≤ 40% is defined as HF with reduced ejection fraction, HF with an ejection fraction ≥ 50% is defined as HF with preserved ejection fraction (HEFpEF), and HF with mildly reduced ejection fraction (HFmrEF) is defined in patients with an ejection fraction falling between 40% and 50 [1]. The number of HF patients is growing rapidly, as 5.7 million individuals in the US suffer from HF; this is worrisome, as more than 8 million people are expected to have HF by 2030, leading to a prevalence increase of 46% [2]. Despite the major advances in the medical treatment for heart failure, with current evidence showing the impact of guideline-directed medical therapy on HF outcome, heart failure remains a leading cause of morbidity and mortality worldwide [3]. Advances in cardiac surgery and biomedical engineering resulted in the creation of new devices that provide short-term or durable, partial or complete replacement of cardiac function [3]. About 70 years ago, mechanically assisted circulation was implicated for ensuring perfusion after cardioplegia for open-heart surgery. Heart transplantation became feasible a few decades later, providing a chance to extend survival to patients with end stage HF [3]. In a randomized clinical trial, an important milestone is represented by demonstrating the superiority of a left ventricular assist device (LVAD) compared to medical therapy in patients with advanced HF who are inotrope-dependent and deemed unsuitable to heart transplantation [3]. Compared to the pulsatile-flow device, which led to an increase of 50% from an expected 1-year survival of implanted patients from 2001 until now, the most recent continuous flow pumps led to an increase of more than 80% in the expected 1-year survival [3]. Improved clinical results and outcomes in terms of survival, hospitalizations for device-related complications, and quality of life have been obtained with the use of continuous flow device–left ventricular devices (CF-LVAD) despite the similarity of the total artificial heart or biventricular support and pulsatile flow to the native heart physiology [3].

The aim of this paper is to analyze the effectiveness, challenges and advancements in the field of LVADs in the treatment of advanced heart failure and to focus on the clinical outcomes with LVADs, including the survival rates and quality of life in addition to highlighting the technological and biomedical advances associated with LVADs.

## 2. Pathophysiology of Heart Failure Leading to LVAD Implementation

According to the American College of Cardiology (ACC)/American Heart Association (AHA), patients with advanced heart failure are those who evolve unfavorably, with a progressive worsening of symptoms that are disabling for daily life, refractory to all therapies. These patients may require advanced therapy such as long-term mechanical circulatory support (LT-MCS), heart transplantation (HT), or may require palliative therapies (e.g., ultrafiltration, intermittent inotropic infusion, peritoneal dialysis to control congestion or end-of-life comfort care) [4].

## 3. Criteria and Symptoms Useful in Defining Advanced HF

Advanced heart failure is defined according to several criteria and symptoms as shown in Table 1. Additionally, in order to classify patients to be considered for long-term MCS device implantation, the Interagency Registry for Mechanically Assisted Circulatory Support (INTERMACS) profiles were developed as seen in Table 2, taking into consideration the hemodynamic compromise, symptoms and characteristics consistent with a need for advanced therapies. The classification includes seven profiles arranged from shorter to longer recommended maximum time frame for intervention and from highest to lowest clinical severity [4].

The following list provides a detailed description of each profile and the recommended timing of different interventions:

**Table 1:** Criteria and symptoms defining advanced heart failure.

Criteria	Description	Symptoms	Clinical Relevance
Ejection Fraction (EF)	Reduced EF (≤40%) indicative of heart pump failure	Severe fatigue	Poor prognosis; reflects systolic dysfunction
		Shortness of breath (dyspnea)	Impacts quality of life and exercise capacity
		Orthopnea	Indicates worsening congestion
		Paroxysmal nocturnal dyspnea	Suggests fluid overload during sleep
		Swelling (edema)	Reflects systemic venous congestion
NYHA Functional Classification	Class III: Marked limitation of activity	Decreased exercise tolerance	Guides treatment decisions and prognosis
	Class IV: Symptoms at rest	Cough or wheezing	May indicate pulmonary congestion
Biomarkers	Elevated B-type natriuretic peptide (BNP)	Rapid or irregular heartbeat	Higher levels correlate with severity
	Indicates heart stress and fluid overload	Confusion or difficulty concentrating	Suggests reduced cardiac output
Clinical Signs	Jugular venous distension (JVD)	Cold extremities	Indicates poor peripheral perfusion
	Peripheral edema	Cyanosis	Suggests severe heart failure
	Ascites (fluid in the abdomen)	Signs of worsening renal function	Renal impairment often accompanies advanced HF
Hospitalization History	Frequent hospitalizations for HF within the past year	Increased need for diuretics	Reflects worsening disease trajectory
	Indicates instability and high resource utilization		High risk of acute decompensation

**Table 2:** The Interagency Registry for Mechanically Assisted Circulatory Support (INTERMACS) profiles.

	Profile Description
Profile 1	Critical cardiogenic shock with severe end-organ dysfunction (inotrope dependent, mechanical ventilation, or circulatory support required). Urgent need for transplantation or LVAD.
Profile 2	Progressive decline despite maximal medical therapy. Frequent hospitalizations for heart failure exacerbations. Inotrope-dependent but stable for transplantation or LVAD.
Profile 3	Stable on inotropes or mild outpatient support. Limited physical activity due to heart failure symptoms. Ambulatory and awaiting transplant or LVAD.
Profile 4	Ambulatory with severe symptoms. Unable to perform most activities of daily living due to heart failure symptoms. Requiring minimal inotropic support.
Profile 5	Ambulatory but with moderate symptoms. Comfortable at rest but limited with exertion.
Profile 6	Ambulatory with mild symptoms. Symptoms are not limiting.
Profile 7	Advanced NYHA Class IV but stable with optimal medical therapy. No symptoms at rest.

## 4. Laboratory Evaluation

Hematological workup is recommended for LVAD candidates due to bleeding risk post-LVAD implantation, which is multifactorial and has been linked to hepatic dysfunction, anticoagulation, fibrinolysis, renal impairment and anti-platelet therapy [5]. Platelet dysfunction and impaired von Willebrand factor activity are LVAD-induced major acquired coagulopathy [5]. It is important to assess for thrombocytopenia, iron deficiency, heparin-induced thrombocytopenia, and hypercoagulable states preoperatively, as these have been associated with higher risk of adverse events post-LVAD implantation [5].

Renal evaluation is essential preoperatively since worse outcomes are seen in patients with pre-implant glomerular filtration rate (GFR) under 30 mL/min/m^2^ compared with those with GFRs greater than 30 mL/min/m^2^ [6]. Diabetes, proteinuria, hepatic dysfunction, and intraoperative blood product use are associated with worse outcomes post-LVAD [5]. End-stage renal disease and dialysis for LVAD recipients are also associated with poor prognosis [6]. Patients who are already on chronic dialysis prior to implantation are not considered for LVAD placement as destination therapy [6].

Poor outcomes after implantation such as neurological events, postoperative major bleeding and platelet dysfunction are encountered in patients with pre-existing liver dysfunction [5]. A valuable screening tool known as the Model for End-Stage Liver Disease (MELD) has emerged in addition to complete metabolic panel to assess for aminotransferases, bilirubin, and albumin as part of the laboratory testing in routine evaluation [5]. Increased risks of bleeding, renal failure, right ventricular (RV) failure, and device infection post-LVAD implantation have been associated with a high MELD score, calculated from creatinine, international normalized ratio (INR) and bilirubin [5]. A viable alternative to assess liver function in HF patients on anticoagulation is the MELD-XI score, which excludes the INR. Poor outcomes post-LVAD implantation are associated with scores of MELD and MELD-XI above 17, thus serving as a useful tool to assess preoperative implant risk [5].

Echocardiography examination is important to assess the RV function prior to LVAD implantation, as significant morbidity and mortality have been associated with RV dysfunction. Risk for RV failure can be assessed by echocardiographic parameters in combination with invasive right-heart catheterization by measuring four important parameters. The right atrial pressure reflects the central venous pressure, the pulmonary arterial pressure, the pulmonary arterial wedge pressure, and the cardiac output, in addition to calculating the pulmonary pulsatility index, which is predictive of RV failure post-LVAD implantation [5]. Smaller pre-implant ventricular size has resulted in worsened post-LVAD outcome, so evaluation of left ventricular dimension is also important [5]. In addition, diagnosis of aortic regurgitation, mitral stenosis, pulmonic valve stenosis, or tricuspid valve stenosis by echocardiography is essential since surgical intervention by the time of LVAD placement is required, as aortic regurgitation becomes worse after the implantation of the LVAD, leading to significant hemodynamic consequences [5].

## 5. First-Generation LVAD

**Figure 1:**
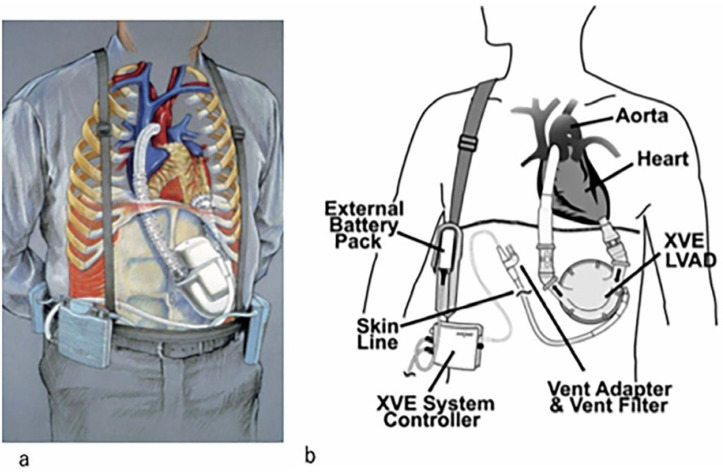
First generation implantable left ventricular assist device (pulsatile devices). (**a**) Novacor N100, (**b**) HeartMate XVE. Adapted from “Review of Implantable Left Ventricular Assist Devices” by E. Tayama, K. Takagi, T. Shojima, H. Otsuka, T. Takaseya, & K. Arinaga, 2023, Kurume Medical Journal, 68(3.4), p. 176. CC BY-NC [7].

As shown in Figure 1 (Tayama et al., 2023 [7]), the first-generation implantable pulsatile LVAD appeared in the 1990s. HeartMate devices were first used [7]. While the pump itself is placed in the intraperitoneal or preperitoneal space, the blood in these devices is pumped from the apex of the left ventricle to the ascending aorta. Connected to the pump and a system controller outside the body and the electric power source via a subcutaneous tunnel is a drive line supplying electric power [7]. This provided electrical power for 3–5 h. Because the side of the pump was made of a textured surface with titanium alloy microbeads, the HeartMate pulsatile VAD showed excellent antithrombotic properties [7]. Individuals with small physique are not candidates for the first-generation LVAD owing to the extremely large pump size [7].

## 6. Second-Generation LVAD

**Figure 2:**
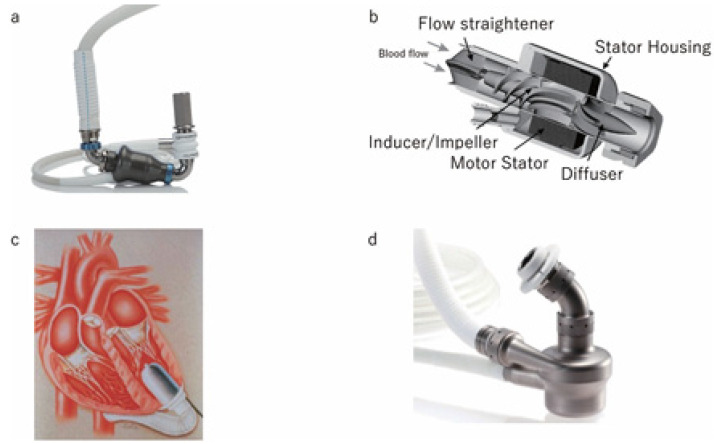
Second-generation implantable left ventricular assist devices (continuous flow devices). (**a**) HeartMate II, the most widely used LVAD in the world. (**b**) Micromed DeBakey VAD, the first clinically applied flow pump in the world. (**c**) Jarvik 2000 an extremely small axial flow pump that reduce driveline infection by fixing the driveline at the posterior skull of the auricle. (**d**) EVAHEART, a centrifugal pump developed in Japan, rotates in a non-contact manner by circulating water around the rotating shaft improving the antithrombotic properties around the shaft. Adapted from “Review of Implantable Left Ventricular Assist Devices” by E. Tayama, K. Takagi, T. Shojima, H. Otsuka, T. Takaseya, & K. Arinaga, 2023, Kurume Medical Journal, 68(3.4), p. 175. CC BY-NC [7].

As shown in Figure 2 (Tayama et al., 2023 [7]), in order to overcome the limitations of the first-generation pulsatile pumps, such as excessive device size, poor mechanical reliability, and high manufacturing cost, second-generation LVADs known as continuous flow pumps were used [7]. The axial flow pump and a centrifugal pump are the two distinct designs that emerged for the continuous flow pump. The centrifugal flow was generated by a bladed disk spinning in a cavity in the EVERHEART (Figure 2d), while the axial flow is generated by propeller in a pipe. Typical devices were the HeartMate II (Figure 2a), Micromed DeBakey (Figure 2b), Abbott Park, and Jarvik 2000 (Figure 2c) [7]. The Jarvik 2000 is implanted in a true intraventricular position without the need for an inflow component, weighing only 90gr and functioning as a continuous flow LVAD [7]. The simple structure of the continuous flow pump comprises moving parts without a valve, with the risk of pump failure and manufacturing cost being lower than those of pulsatile pumps [7]. Easier implantation in the abdominal wall or thoracic/abdominal cavity is due to the relatively small pump size compared to the pulsatile device. Similar to the first-generation implantable VADs, the second-generation devices are connected to an external power source via a driveline [7]. Long-term organ damage can be seen in continuous flow devices due to its non-physiological non-pulsatile flow, but many benefits can be achieved by reducing the device size and increasing its durability, which is believed to be approximately 5 years [7].

## 7. Third-Generation Left Ventricular Assist Device

A third-generation LVAD was developed to further improve device durability and reliability where the bearing mechanism is the major difference between the second- and the third-generation devices [7]. In order to reduce shear and prolong LVAD durability, the third-generation LVADs had a moving impeller that is suspended by magnetic and/or hydrodynamic forces [7]. The HeartMate III LVAD has a considerable decrease in all dimensions from its predecessor, with a weight of 200 g and a diameter of 50.3 mm and a height of 55.8 mm. The HeartMate III is a centrifugal-flow device utilizing bearingless full magnetic levitation and is fully implantable in the thoracic cavity in contrast to HeartMate II, which has continuous axial flow and is fully reliant on mechanical bearings. A short 20 mm inflow cannula implanted directly into the LV apex allows blood to be received by the pump housing, and the ejection of blood occurs via a 14 mm graft connecting the pump housing and the ascending aorta [8].

Less sheer stress and red blood cell destruction is encountered compared to earlier designs due to the fully magnetically levitated design, which allows for wide gaps between the impeller and elements of the pump housing combined with the flat surfaces of the pump’s interface [8]. As a result, less pathologic bleeding is encountered because there is less multimer degradation of von Willebrand’s factor, which is supported by lower circulating levels of lactate dehydrogenase in HM3 patients, indicating less hemolysis [8]. Thrombus formation is therefore minimized due the frictionless environment and the wide passages of this system [8].

## 8. LVAD Indications and Patient Selection

There must be clear guidelines for selecting patients for LVAD implantation. Some key criteria are listed in Table 3. First, guidelines presented by the American Association for Thoracic Surgery (AATS) and the International Society for Heart and Lung Transplantation (ISHLT) emphasize thorough preoperative evaluation for ideal management. Specifically, candidates who are listed for heart transplantation but likely to deteriorate before a donor heart becomes available are eligible for Bridge to Transplant (BTT), given that these patients are likely to survive with mechanical circulatory support (MCS) until transplantation. Candidates who are expected to recover sufficient myocardial function may benefit from Bridge to Recovery (BTR) treatment, as in cases of myocarditis or peripartum cardiomyopathy. Patients who are not candidates for heart transplantation but are expected to benefit from long-term MCS may receive destination therapy (DT), whereby the LVAD is used as a permanent treatment rather than a bridge to transplantation. It is important that these patients maintain an acceptable quality of life and remain compliant with the post-implant care [9].

Patients considered for LVAD implantation should generally exhibit reasonable overall health beyond their cardiac condition, including adequate kidney and liver function, absence of severe infections, and no other medical conditions that could substantially impact the success of LVAD implantation and subsequent recovery [5]. Consequently, patients undergo a battery of tests to evaluate heart function, including echocardiograms, cardiac catheterizations, and other imaging studies to ascertain the extent of heart failure and the potential for recovery [5]. Patient selection for LVADs is increasingly critical due to the shortage of donor hearts. A comprehensive multiparametric assessment is necessary to determine candidacy, evaluating baseline status, frailty, comorbidities including renal and hepatic dysfunction, and medical history, encompassing all prior cardiac conditions [10]. These comorbid conditions should be managed before and throughout device implantation [11]. Alongside comorbidities, the patient’s mental health, social support, and ability to adhere to post-implant care must also be taken into consideration. Discussions must also be made regarding the patient’s values, goals, and understanding of the risks and benefits of MCS (AACTS/ISHLT) [9].

**Table 3:** Summary of LVAD indications and patient selection criteria.

Indication	Criteria	Source
Bridge to Transplant (BTT)	Recommended for those listed for heart transplantation who are expected to decline before availability. The patients are likely to survive with MCS.	AATS, ISHLT [9]
Bridge to Recovery (BTR)	Myocarditis, peripartum cardiomyopathy, or other conditions where myocardial recovery is anticipated.	AATS, ISHLT [9]
Destination Therapy (DT)	Recommended for patients ineligible for heart transplantation but expected to benefit from long-term MCS, with preserved quality of life and compliance with post-implant care.	AATS, ISHLT [9]
General Health Requirements	Patients must have adequate kidney and liver function, absence of severe infections, good overall health beyond HF, stable mental health, and good social support.	ACC/AHA [11], ESC [12]
Comorbidities Considerations	Manage renal and hepatic dysfunction, frailty, and prior cardiac conditions. Avoid reversible heart failure causes and contraindications.	AATS, ESC [12], EACTS [5], ACC/AHA [11]
Advanced Heart Failure	NYHA Class III-IV, refractory to guideline-directed medical therapy, EF < 25%, stage D HF, and recurrent hospitalizations.	ESC [12], ACC/AHA [11], AACTS/ISHLT [9]
Hemodynamic Assessment	Assessment of cardiac output, pulmonary artery pressure, and lack of contraindications.	ESC [12]
Risk Stratification	INTERMACS 2–4, inotrope dependence, progressive end-organ dysfunction, peak VO2 < 12mL/Kg/min, temporary MCS dependence, or complications from previous treatments.	ESC [12], EACTS [5]
Special Considerations	Elevated pulmonary vascular resistance, renal failure, recent cancer, obesity, drug or alcohol recovery, multiple HF hospitalizations, hypotension, and sustained ventricular arrythmias	EACTS [5], ACC/AHA [11], ESC [12]

According to the ESC 2021 guidelines, LVAD candidates consist of patients suffering from advanced heart failure symptoms, including limitations in physical activity and/or severe symptoms at rest (NYHA Class III or IV) despite receiving optimal medical therapy [12] and AACTS/ISHLT [9]. In this sense, patients should exhibit symptoms that are refractory to guideline-directed medical therapy, including angiotensin-converting enzyme inhibitors (ACEi), beta-blockers, mineralocorticoid receptor antagonists, and sodium-glucose co-transporter-2 inhibitors (SGLT2i), among others [12]. These candidates typically have a significantly reduced left ventricular ejection fraction (LVEF) of <25% [12] + ACC/AHA [11]. To ensure proper patient selection, the ESC recommends assessment of hemodynamic profile, such as cardiac output and pulmonary artery pressure, and ensuring the lack of contraindications [12]. In terms of ACC/AHA staging, stage D HF is characterized by severe refractory symptoms of HF despite guideline-directed medical therapy (GDMT). These symptoms interfere with daily life and lead to recurrent hospitalizations. Therefore, LVAD therapy is reserved for patients with stage D heart failure [12]. Certainly, a multidisciplinary team comprising cardiologists, surgeons, nurses, and other healthcare professionals must collaborate in the decision-making process [13]. Nonetheless, one must note that a crucial window exists where implantation is neither too early to risk complications nor too late to cause multiorgan dysfunction [6].

The 2019 European Association for Cardio-Thoracic Surgery (EACTS) guidelines give a class I recommendation that patients with reversible causes of HF should be ruled out from LT-MCS [6]. LT-MCS implantation is a class IIa recommendation in patients with NYHA functional class IIIB-IV and EF ≤ 25% and at least one of the following criteria: INTERMACS 2–4, inotrope dependence, progressive end-organ dysfunction, peak VO2 < 12 mL/kg/min, or temporary MCS dependence [6]. LT-MCS implantation is a class IIb recommendation in patients with NYHA functional class IIIB-IV and EF ≤ 25% who would benefit from reversing elevated pulmonary vascular resistance or renal failure, or in patients with recent cancer, obesity, or recovering drug and alcohol dependence [6].

Individuals with two or more heart failure hospitalizations in the last 12 months, intolerance to guideline-directed medical therapy due to symptomatic hypotension, inotrope dependence, high or escalating diuretic doses, and multiple episodes of sustained ventricular arrhythmias must be evaluated [6]. Innovative methods for risk stratification and management of cardiac and non-cardiac comorbidities can lead to improved outcomes in LVAD recipients. Incorporating quality of life metrics and measurements of adverse events can better inform heart failure cardiologists to identify ideal LVAD candidates. Moreover, the integration of machine learning algorithms may guide patient selection to enhance outcomes. Hence, patient selection and assessment of reversible medical comorbidities are crucial to the postoperative success of LVAD implantation. Identifying patients most likely to benefit and least likely to experience adverse events should be a priority in patient selection for LVADs [14].

## 9. Surgical Approach

a. Median Sternotomy

The surgical procedure for implantation of LVAD is almost similar in all types of LVAD. A typical median sternotomy is carried out. Before the pericardium reaches the diaphragm, it is cut and divided to the left of the midline for a few millimeters, and it is then squared off in both directions [15]. The left side of the dissection continues a few centimeters above the phrenic nerve, exposing the heart’s apex. Owing to the HeartMate II pump’s dimensions and design, an input and outflow cannula are positioned in a preperitoneal pocket to maximize flow and prevent compression of either ventricle [15]. A careful dissection of subcutaneous tissue through the incision beyond the diaphragm is necessary, with the incision made anterior to the posterior rectus sheath [15].

A pump pocket that extends 7 to 10 cm below the xiphoid process and is deep enough to extend laterally under the costal arch is considered optimum [15]. Dissection proceeds without going past the xiphoid and preserves the integrity of a closed left pleural space thanks to the lower-profile HeartMate 3 and Heartware Ventricular Assist Device (HVAD) [15]. This dissection and tunneling of the driveline are usually performed in all patients before the heparin is given [15].

b. The driveline

The percutaneous driveline’s location depends on the anatomy and preferences of the patient [15]. The placement is intended to prevent line fractures and/or ascending infection, as well as to avoid interfering with pannus folds and beltlines [15]. All three gadgets’ drivelines are silicone-coated wires with a felt-covered section [15]. When positioned properly, the silicone covering the driveline lowers issues related to line breakage and malfunction while also acting as a barrier against moisture for the cable [15]. The driveline’s velour or felt section, which is meant to remain inside the subcutaneous tissue, provides stability by producing an adherent effect that adheres to internal surfaces [15]. Thus, the location of the driveline that is advised is referred to as a silicone-to-skin interface into the use of a tunneling instrument; the driveline is positioned into the rectus muscle along the anterior axillary line, about 2 cm below the costal border on either side [15]. The driveline does not enter the peritoneal area; instead, it passes through subcutaneous tissue [15]. After that, it emerges through a skin incision at the designated spot, with the felt buried one to two centimeters away from the exit [15]. Other methods that have been reported include the double-tunnel approach and the rectus-sparing technique, which both require a longer driveline passage in the subcutaneous tissues of the preperitoneal area without a pericardial loop [15]. The goal is to lessen the chance of ascending infection to the mediastinum or pump pocket [15].

c. Cannulation and cardiopulmonary bypass

A bypass is planned by placing cannulation sutures following the delivery of systemic heparin [15]. Surgeon preference determines the approach for either dual-stage or bicaval cannulation for venous drainage, aside from typical arterial access in the aorta [15]. The latter is required if a patent foramen ovale is found and has to be closed, or if issues with the mitral or tricuspid valves need to be resolved [15]. It is necessary to provide space for an aortic root vent in addition to the outflow graft when positioning the aortic cannula [15]. Pledged cannulation sutures and needless dissection are avoided whenever possible in preparation for a potential future reentry [15]. After going on bypass, the heart begins to beat more softly, and the apical dimple next to the distal left anterior descending artery is clearly visible [15]. Transesophageal echocardiography (TEE) is used to guide decisions on whether to enter here or 1 to 2 cm lateral to the apical dimple in order to make sure the inflow cannula is pointed correctly in the direction of the mitral valve [15]. After marking, moist laparotomy pads are positioned on the diaphragm posteriorly to keep the apex high for further viewing [15].

d. Inflow

The position of the inflow cannula is crucial for the pump’s long-term success [15]. According to the EACTS 2019 guidelines, inflow cannula placement into the LV is recommended and TEE is used to check its position [6]. Furthermore, the inflow cannula should be placed parallel to the septum. Additionally, placing the cannula in the inferior left ventricular wall may be considered [6]. The coring device must be inserted and deployed with equal distribution of surrounding muscle to ensure proper orientation [15]. The apex is cored before or after the placement of ring sutures, with surgeon preference [15]. In the HeartMate II, a VICRYL, 0 suture is placed in the center of the marked area, fed through the coring device, and held with slight tension while gently twisting it into the apex in a perpendicular direction [15]. Individual pledgeted vertical mattress sutures ensure adequate hemostasis around the sewing ring. In the HeartMate 3 and HVAD, the sewing ring is held against the predetermined location on the LV’s distal anterior surface, and individual pledgeted sutures are placed deep into the myocardium [15]. The myocardium is incised in the center of the sewing ring, and the punch used to remove the core is used. Careful deployment of the coring device ensures a properly placed pump [15]. The inlet cannula is inserted into the LV after the sutures are secured and the ring is secure [15]. The pump has a specific point of attachment that requires appropriate positioning [15]. The HVAD has a screw directed towards the heart’s base close to and parallel to the left anterior descending artery to assess the pump [15]. The HeartMate 3 uses a slide lock locking mechanism, which is retracted to be opened before inserting the pump in the LV [15]. The pump is rotated to direct the outflow graft toward the right ventricle and the driveline toward the midline [15]. The slide lock is engaged when the yellow zone inside is no longer visible [15]. This lock is beneficial if the pump requires reorientation, as it ensures a sterile surgical instrument is used [15].

e. Outflow graft

The cannula should ideally be positioned in the atrioventricular groove, along the right atrial gutter, and gently curve around the acute border [15]. The EACTS 2019 guidelines recommend positioning the outflow graft along the inferior right ventricular surface and between the right atrium and pericardium to avoid crossing the right ventricular outflow tract [6]. This might be measured by expanding the graft by partially filling the LV. Before cutting, the outflow graft should be stretched to determine the proper length. After the size is established, a vascular clamp is used to occlude the graft, and the LV is drained in preparation for the aorta to be fully connected [15].

Based on the EACTS 2019 guidelines, performing the outflow graft anastomosis on the ascending aorta at a 45 degree angle is recommended [6]. This can be performed by using a partial occluding clamp and threading a 4-0 or 5-0 polypropylene suture in a running fashion into a beveled graft, beginning at the heel [15]. The optimal location is distal to the sinotubular junction, on the lateral side of the proximal ascending aorta’s larger curvature [15]. After placing the clamp, a knife is used to create an aortotomy, which is then stretched using a punch instrument [15]. First, palpate the aorta to prevent entering a calcified plaque [15]. To prevent narrowing of the anastomosis and clear visualization of the back wall for strategically placed sutures, make the incision wide enough to accommodate the appropriate cannula [15]. After completing the anastomosis, the outflow graft’s vascular clamp is momentarily withdrawn to remove air from the system before being reintroduced [15].

f. Less invasive approach

Less invasive centrifugal VAD implantation can be performed by two main techniques: hemi-sternotomy and left antero-lateral thoracotomy approach, and sternum sparing technique with one right mini-thoracotomy incision (traditionally second intercostal space) and another left antero-lateral thoracotomy incision [16]. The procedure is similar for both techniques, except for a few differences:

A. For ideal exposure for the thoracotomy, place the patient supine and rotated 30 degrees to the right.
B. Prepare the sterile field as for a full sternotomy.
C. 
  a. Perform a J-shaped upper hemi-sternotomy up to the second and third intercostal spaces based on the position of the aorta. The inverted Y technique has been described. However, it was shown that this technique decreases chest wall stability after aortic valve replacement surgery.
  b. Perform an incision at the right second intercostal space to achieve outflow graft anastomosis in the sternum sparing technique.
D. Left antero-lateral thoracotomy is common in both techniques; TTE guidance is used to localize the exact location of the thoracotomy.
E. Correctly localize the spot for LV core by poking a finger into the LV apex and confirm the position using a transthoracic echocardiogram (TTE).
F. Suture the inflow sewing ring to the heart without the cardiopulmonary bypass (CPB) machine.
G. Heparinize the patient and start CPB using venous cannulation into the right femoral vein and arterial cannulation into the ascending aorta; in the sternum sparing approach, cannulation of the vessels is essential.
H. Core the LV apex to place the pump and inspect the LV for thrombus and trabeculae.
I. Place and secure the pump.
J. 
  a. If no previous sternotomy is performed, tunnel the outflow graft intra-pericardially, lateral to the right ventricle towards the ascending aorta through a pericardial incision close to the aortic base.
  b. If a previous sternotomy is performed, tunneling can be performed extra-pericardially, medial to the left lung, in order to avoid laceration of cardiac structures (particularly in the presence of open LIMA-LAD bypass) due to expected adhesions.
K. Using a surgical forceps, insert an incision and bind the free end of the outflow graft with thick silk suture, then grasp and pull it towards the aorta.
L. Tunnel the driveline using “a dual-incision modified long subfascial C-shaped technique also known as double tunnel technique”. This approach was shown to decrease the rate of infection.
M. Close the pericardial layers at the end of the procedure [16].

## 10. Post-Operative Care

The main goal in post-operative care of LVAD patients is to maintain a good tissue perfusion as well as achieve hemostasis and support the RV function [17]. Perfusion is maintained by balancing cardiac output and systemic vascular resistance by a combination of vasopressors and inotropes to obtain a mean arterial pressure (MAP) of 60–70 mmHg and cardiac index > 2.2 [17]. The preferred combination is noradrenaline and dobutamine. Additional measures are taken regarding other organ systems [17]. According to the EACTS 2019 guidelines, several post-operative measures must be taken including [6]:

- Goal MAP: 60–70 mmHg.
- Avoid MAP >85 mmHg due to risk of reduction in LVAD flow and bleeding.
- Monitoring using continuous electrocardiography, pulse oximetry, central venous pressure and invasive arterial blood pressure is recommended [6].
- Daily measurement of plasma free hemoglobin and lactate dehydrogenase is recommended [6].
- Inotropes for hours or days is beneficial, as it helps the RV adapt to its new loading parameters. Milrinone, dobutamine, and levosimendan are first-line agents. Epinephrine might be used as first line in case of severe hypotension. Combining norepinephrine and dobutamine instead of epinephrine can be considered in case of postoperative hypotension and low cardiac output syndrome with RV failure [6].
- Vasoactive drug dosage should be adjusted to maintain a central venous pressure of 5 to 15 mmHg and a pulmonary arterial wedge pressure less than 15 mmHg. Nitric oxide or sildenafil can be used to treat pulmonary hypertension [18].
- Endotracheal tubes with subglottic secretion drainage systems can reduce microaspiration of oropharyngeal flora. This procedure was found to decrease ventilator-associated pneumonia [17].
- Measures to prevent pulmonary complications include incentive spirometry with deep breathing techniques, directed coughing, early mobilization, and optimal analgesia.
- Improve mucocilliary clearance in early post-extubation phase using bronchodilators such as albuterol, stimulating productive cough, and reducing sputum viscoelasticity using hypertonic saline aerosols. However, these aerosols are contraindicated in COPD patients due to airway irritation [17].
- It is recommended to avoid hypercarbia to prevent the increase in pulmonary artery pressure and RV afterload [6].
- Maintaining miniaturized TEE probes in the esophagus in situ for up to 72 h to help in the management of fluid resuscitation and to diagnose complication [6].
- Echocardiography is recommended to guide weaning from temporary RV support [6].
- Inhaled NO, epoprostenol (or prostacyclin) and phosphodiesterase 5 inhibitors (PDEis) may be used to reduce right heart failure after LVAD implantation [6].
- Continuous monitoring of the flow rate of the blood pump for any abnormal change because the patient is at risk of bleeding following anticoagulation therapy.
- Anticoagulation is essential and can be started 8 h post-surgery if bleeding is <50 mL/h. Unfractionated heparin is commonly used as well as direct thrombin inhibitors that have been reported to be successful. Initial target of partial thromboplastin time (PTT) is 40 s, which is increased progressively within the 48–72 h post-operatively to 55–60 s. Oral vitamin K antagonist should be initiated once the patient is stable and oral intake is possible. The target INR is dependent on the device recommendations for modern LT-MCS devices. The INR target is between 2.0 and 3.0 and bridging with intravenous heparin is recommended if the INR is <2.0 and in cases of planned invasive procedures or non-cardiac surgical procedures for perioperative bridging. Low-molecular-weight heparin may be considered as well. The use of acetylsalicylic acid is also recommended. In case of bleeding episodes, it is recommended to re-evaluate the antithrombotic therapy [6].
- Monitor the patient for neurological signs and symptoms such as impaired consciousness, and focal neurological deficit.
- Surgical re-exploration is considered if mediastinal drainage exceeds 150–200 mL/h during the early postoperative phase [6].
- Cardiac rehabilitation and exercise training are recommended as Class I evidence in recent trials and cardiovascular disease societies. It starts by early daily active/passive bicycle training in the bedside recumbent position to improve muscle strength, followed by gradual transition to bedside standing, bedside stepping training, assisted walking, and finally independent walking [18].

## 11. Complications and Management

According to a study carried out by Adesiyun et al. (2017), LVAD-related complications developed in 74% of the cohort during follow-up. The most common complications were driveline infection (42%), anemia requiring transfusion (32%), and sepsis or bacteremia (24%), GI bleed (22%) and CVAs (18%) [19]. Sepsis was confirmed by positive blood cultures and meeting the systemic inflammatory response criteria, bacteremia by positive blood cultures, gastrointestinal (GI) bleeding by drop in hematocrit and positive endoscopy or stool guaiac, driveline infection by positive cultures from the drive line site, and embolic and hemorrhagic cerebrovascular accidents (CVAs) [19]. The study has found that CVA and sepsis or bacteremia were important predictors of mortality [19]. CVA was observed after a median of 217 days (IQR 95-531) and sepsis after a median of 454 days (IQR 146-622) [19].

The most common complications are the following:

a. Pump thrombosis

According to Phan et al. (2023), a multivariable analysis identified specific factors that contribute to pump thrombosis. These included high mean arterial blood pressure, poor anticoagulation and antiplatelet control in addition to elevated lactate dehydrogenase (LDH) [20]. Device-related factors include cannula malposition and alignment, and have been associated with increased risk for pump thrombosis due to “flow disturbance secondary to deviations of the inflow cannula from the mitral-apical axis” [20]. A significant modifiable risk factor for pump thrombosis has been identified as arrhythmias, such as atrial fibrillation, because of the elevated risk of stroke and thromboembolic events [20].

Increased LVAD power values (>30%), isolated elevation of LDH levels > 3 times the usual values, and symptoms of newly formed heart failure without underlying etiology are all indicative of hemolysis due to pump thrombosis [20]. Additional clinical indications and symptoms, such as worsening heart failure symptoms, an LVAD low-flow warning or greater pump power, free hemoglobin > 40 g/dL, and LDH > 800 IU/L can help diagnose LVAD pump thrombosis [20]. Moreover, posterior rotation of the input cannula, which may be a risk factor for the development of pump thrombosis, can be detected by imaging techniques such as CT and plain radiographs [20].

Three distinct LVAD sites are susceptible to pump thrombosis: the outflow graft, the pump itself (often between the impeller and the pump housing), or the inflow cannula (Table 4) [21]. Suspicion of outflow graft blockage or stenosis usually arises when the device shows a drop in power and flow rates (Table 4), but maintains a constant speed (rpm) [21]. On the other hand, a rise in power and flow rates typically points to a possible inherent problem with the apparatus [21]. HeartMate III LVADs have a lower incidence of pump thrombosis than previous generation devices, but this is still a significant clinical problem that requires early detection [21]. Cardiac Computed Tomography Angiography (CCTA) is a crucial diagnostic tool to locate a thrombus [21]. Takla et al. (2024) present a patient with recurrent low-flow alarms that raised concerns for the possibility of device obstruction or thrombosis. This has a high risk of morbidity and mortality and increased risk of stroke and pump failure (Table 4) [21]. Gated CCTA was performed and showed focal narrowing of the LVAD outflow graft caused by focal kinking and an eccentric thrombus [21].

A micropuncture needle was used to access the right common femoral artery under ultrasound guidance [21]. A micropuncture wire and micropuncture sheath were then advanced [21]. A 7F sheath was inserted after a J-wire was pushed into the aorta [21]. Next, the wire was removed and the right common femoral artery received two preplaced Perclose ProGlideTM devices from Abbott Vascular [21]. A total of 10,000 units of heparin were administered systemically to the patient while keeping their active clotting time greater than 250 s [21]. Next, an 8.8 F Terumo NagareTM sheath was advanced in the proximal descending thoracic aorta and an exchange-length Glidewire Advantage (Terumo International Systems) was pushed into the ascending aorta [21]. An aortography was carried out [21]. The LVAD outflow limb opening in the descending thoracic aorta was then engaged by angulating the steerable sheath [21]. The stenosis was traversed and the outflow limb was cannulated using the exchange-length Glidewire Advantage in conjunction with a 135 angled Terumo NaviCross catheter, maintaining access to the proximal LVAD outflow limb close to the LVAD [21].

In order to prevent pump thrombosis, patients need to be placed on both aspirin and warfarin to target an INR of 2–3, after utilizing a heparin bridge [21]. In addition to anticoagulation, IV fluid administration and alkalization of the urine using sodium bicarbonate drips should be performed to prevent Acute Kidney Injury (AKI) secondary to hemolysis of red blood cells [21]. If the patient is unstable and refractory to heparin, a pump exchange is indicated [21]. Heart transplantation is the last definitive therapy for the prevention of LVAD pump thrombosis [21].

A study found that 14 out of 94 patients experienced a pump thrombosis after LVAD implantation (11%) and systemic thrombolysis was successful in 10 of 14 patients (71%) at 30 days [23]. A combination of phenprocoumon and antiplatelet therapy was used [23].

For the treatment of LVAD thrombosis, a variety of percutaneous clot dissolving techniques, such as catheter-directed thrombolytic infusion, rheolytic thrombectomy, mechanical fragmentation, ultrasonic fragmentation, or thrombus aspiration, may be used [22]. Nonetheless, extreme caution should be used during percutaneous thrombectomy to prevent the catheter from getting tangled in the LVAD impeller [22]. Although thrombolytic therapy has been approved for the treatment of LVAD thrombosis, it has not been subjected to a thorough clinical review in patients with LVAD [22]. In these cases, intracranial hemorrhage is a dangerous concern [22]. A thrombolysis protocol modification resulted in a lower cumulative dose of r-tPA administered [22]. In a patient with recurrent LVAD thrombosis, repeated rounds of low-dose intravenous alteplase (3 mg bolus with 3 mg/h infusion for 5–6 h) effectively dissolved thrombus several times over the course of months [22]. The total intravenous alteplase dose with this approach was never more than 30 mg [22].

Resolution of symptoms, normalization of LVAD parameters, and normalization of laboratory data are expected outcomes after successful thrombolysis [22]. The diagnosis of thrombosis is indirectly confirmed by rapid clinical improvement following thrombolysis [22]. Auscultation should show a smoothening of the LVAD sound during a physical examination [22]. Reduced power consumption, as well as higher estimated blood flow and pulsatility index, should be displayed by the LVAD controller [22]. The patient should start to be hemodynamically stable [22]. Improved left ventricular unloading should be visible on echocardiography, along with low-velocity laminar flow at the outflow graft anastomosis and inflow cannula [22]. Over several days, lab readings may return to normal [22].

b. Hemorrhagic and ischemic stroke:

Stroke is one of the most feared and fatal post-LVAD complications. Hemorrhagic stroke in patients with LVAD are caused by many things, including anticoagulation, hemorrhagic conversion after ischemic stroke, acquired von Willebrand syndrome and rupture of histologically fragile vessels due to the non-physiological continuous pulseless flow [24]. Post-LVAD high blood pressure, high INR, heparin-induced thrombocytopenia, treatment with intra-aortic balloon pump and female sex are considered pre-implant risk factors for hemorrhagic stroke [24]. Patient with LVAD have a variety of hemorrhagic stroke presentations, ranging from mild neurological signs to deep unconsciousness that may be mistaken for circulatory arrest due to the pulseless nature of the continuous flow [24].

Intraparenchymal hemorrhage (IPH) is the most common type of hemorrhagic stroke followed by subarachnoid (SAH), subdural (SDH), and epidural bleeding [20]. IPH causes more neurologic injury and higher 30-day mortality rate than other types of strokes (38% in IPH vs. 0% in SDH, and 29% in SAH) in patients with LVAD [20]. In another study, 283 patients underwent LVAD placement, among which 32 (11%) experienced ICHs [20]. A total of 47% were IPH, 41% SAH and 12% SDH. HVAD was implanted in 38% of patients, HMII in 62% and no patients received an HM 3 [25].

Device type has been shown to affect the incidence of ischemic strokes in patients [26]. According to the INTERMACS report in 2020, first-generation LVAD had a risk of stroke ranging from 20 to 55%, while the newer generation continuous flow LVADs has an incidence of stroke of 6.3% event per patient year (EPPY) during the early postoperative period (within 90 days) and 4.1% EPPY after 90 days from LVAD implantation [26]. CF-LVAD is associated with reduction in endothelial nitric oxide availability compared with healthy controls and pulsatile LVAD patients, resulting in increased arterial stiffness, which is independently associated with a higher risk of stroke [26]. This underlies the importance of pulsatility for perfusion of the peripheral vasculature [26]. Low pulsatility in CF-LVAD patients has been associated with increases in aortic wall thickening and stiffness with reduced aortic compliance compared with healthy patients and pulsatile LVAD patients [26].

To prevent these complications, a retrospective study of 418 patients undergoing HMII implantation proposed the initiation of oral warfarin and aspirin in the immediate post-operative period rather than intravenous heparin bridging, oral aspirin and warfarin [26]. It has shown a lower rate of bleeding complications (18% vs. 32%, *p* = 0.04) without a rise in perioperative ischemic stroke (IS) (3% vs. 5%) or pump thrombosis (2% vs. 3%) [26].

PDEis, usually used for treatment of pulmonary hypertension and right ventricular unloading, have been associated with reduced IS risk in LVAD patients [26]. The goal of therapy in managing hemorrhagic stroke or intra-cranial hemorrhage (ICH) is reduction in excessive BP and reversing coagulopathy [24]. It is advised to reduce MAP below 90 mmHg [24]. Fresh-frozen plasma (FFP), prothrombin complex concentrate (PCC), vitamin K, or a combination are used in the reversal of warfarin [24]. PCC is preferred in neurosurgical emergencies because of its faster effect [24]. Surgical treatment is sometimes required depending on the severity of presentation such as craniotomy with drainage [24]. Platelet administration is avoided in ICH due to adverse outcomes; nonetheless, it might be beneficial in patients undergoing neurosurgical intervention [24]. Full anticoagulation reversal is necessary for LVAD patients undergoing heart transplantation prior to transplantation, and conventional techniques such as FFP and low-dose 3 factor prothrombin complex concentrates (3F-PCCs) are inadequate [27]. Nevertheless, the use of 4F-PCC in perioperative anticoagulant reversal has grown in popularity [28]. Compared to FFP, 4F-PC, which is generated from human plasma, reverses the anticoagulant effects of VKA more quickly and with lower amounts [27]. According to a randomized research, 55% of patients in the 4F-PCC group had a quick INR drop, and 78 (90%) patients in the group were compared to 61 (75%) in the FFP group [27]. However, a 2019 retrospective study raised concerns about the use of 4F-PCC during the perioperative period, since it was found to increase the risk of thromboembolic events in patients receiving it instead of FFP for rapid warfarin reversal [27]. Only in cases when 4-factor PCC is unavailable does the American College of Cardiology advise FFP for the prompt reversal of anticoagulation in the event of significant bleeding [27]. Using PCC instead of the conventional strategy of vitamin K and FFP reduces the requirement for FFP, according to a new single-center retrospective analysis [27].

c. Renal failure:

AKI is a frequent complication of LVAD implantation and increases the risk of mortality and morbidity [28]. The incidence of AKI post-LVAD implantation ranges between 11% and 45%, and has recently increased to 70% [28]. This complication is lethal, especially when associated with pre- and postoperative right-sided ventricular failure (RVF) [28]. Reports suggest that patients with proteinuria pre-LVAD implantation were at a high risk to develop post-operative AKI [28]. Thus, we should consider nephrologic evaluation, including urinalysis and ultrasound prior to the implant for the work-up in patients with significant renal failure [28].

CPB during the surgery has contributed to renal failure by triggering a systemic inflammatory response syndrome, impairing the vasomotor tone, alternating perfusion to kidneys and producing microemboli in renal capillaries [28]. Excessive bleeding of more than 1 L during the surgery is associated with AKI post-LVAD implantation [28]. LVAD surgery carries a risk of 50% of development of vasoplegia either intra- or post-operatively, especially in continuous flow LVADs, and has been associated with post-operative AKI, although this type has shown to be better in terms of survival, decreased incidence of strokes and better device durability [28].

Continuous flow pumps generate a high shear stress that potentially leads to AKI. The pump reaches a high speed of up to 10,000 revolutions/min, leading to lysis of erythrocytes and causing the release of free iron into the bloodstream, which is toxic to the kidneys [28]. It also reduces the oxygen-carrying capacity of erythrocyte, causing tissue hypoxia within the kidneys [28]. It is worth noting that there are currently no treatments or measures that prevent hemolysis [28].

Right ventricular failure post-LVAD causes AKI because of systemic venous congestion and decreased cardiac output [28]. So, we need to employ several measures to manage RVF by early speed optimization of LVAD, prolonged inotropic support, pulmonary vasodilation, diuresis, early RRT and temporary mechanical circulatory support [28].

According to Yalsin et al. (2019), there is a significant increase in the 30-day mortality of 14% to 18%, and 1-year mortality of 29% to 40% in patients with AKI post-LVAD implantation compared to patients without AKI. Therefore, we should monitor CVP and pulmonary artery pressure in the early post-operative phase, when intravenous fluids are administered and define cutoff values because an increase in CVP > 10−14 mm Hg strongly increases the incidence of AKI in cardiac surgery [28]. There has been a link between elevated intra-abdominal pressure and impaired renal function; thus, we need routine measurement of intra-abdominal pressure, especially in case of abdominal distention, ascites, or discomfort [28]. Discontinue nephrotoxic drugs or switch to less toxic agents [28]. If kidney function keeps worsening, RRT—in the form of continuous or intermittent veno-venous hemofiltration—becomes necessary to control volume status and metabolic derangement [28]. Hemodialysis and peritoneal dialysis are applied for patients with LVADs who do not recover renal function; however, the latter has several potential advantages when compared with hemodialysis, including a lower risk for bloodstream infections, a reduced hemodynamic shift, and a home-based logistic [28].

In a randomized trial, vasopressin was shown to be superior to norepinephrine in preventing AKI and vasoplegic shock in post-cardiac surgery [28]. Methylene blue, an NO inhibitor, is used to treat refractory post-operative hypotension [28]. Hydroxocobalamin has also been used in vasoplegic shock post-LVAD implantation [28]. New agents including selective vasopressin 1-alpha agonist and angiotensin II have been used [28].

d. GI bleed:

GI bleeding is the most common complication of LVADs that requires hospital readmission [20]. According to Phan et al. (2023), the Multicenter Study of MagLev Technology in Patients Undergoing Mechanical Circulatory Support Therapy With HM3 (MOMENTUM 3) trial reported a 17% incidence of GI bleeding within 2 years of an HM3 implant in bridge to transplant (BTT) or bridge-to-transplant candidacy (BTC) and 28.7% in destination therapy (DT). It was shown as well that the longer the duration of implantation, the higher the risk of GI with 21%, 27%, and 31% at 1, 3, and 5 years, respectively [20]. Also, the study has shown that the hospital readmission rate for GI bleeding at 60 days post-LVAD implantation was significantly higher (8.7% vs. 2.3%) than heart failure patients without LVAD [20].

LVAD-associated GI bleeding is considered when the patient fulfills the following criteria: The Interagency Registry of Mechanically Assisted Circulatory Support (INTERMACS) criteria for an adverse bleeding event plus one or more of the following manifestations: hematemesis, melena, or hematochezia [20]. INTERMACS criteria are defined by any incidence of bleeding that results in a hemoglobin drop of greater than 3 g/dL and requires hospitalization, transfusion of packed red blood cells (pRBCs), surgical intervention, intravenous vasoactive agents, or that results in death [20]. The bleeding is most commonly melena (39%), followed by hematochezia (32%) and occult bleeding (24%) [20].

For management of GI bleed, the European Society of Gastrointestinal Endoscopy (ESGE) guidelines recommend red blood cell transfusion to maintain a hemoglobin level between 7 and 9 g/dL, blood pressure monitoring, and fluid resuscitation with crystalloid fluids [20]. Vitamin K antagonists, direct oral anticoagulation, and antiplatelets are withheld until hemostasis is achieved for patients with active bleeding [20].

Octreotide has been the treatment of choice for GI bleed in LVAD patients, as multiple studies demonstrated that it decreases the need of pRBC and FFP transfusion, reduces the length of hospitalization, limits blood pressure in the portal venous system secondary to vasodilation, increases adhesion of platelets and prevents angiogenesis [20]. Several other drugs have shown potential in decreasing GI bleed, including danazol, digoxin and thalidomide [20].

e. Infections:

Infections post-LVAD can be classified into three categories: VAD-specific, VAD-related, and non-VAD [29]. VAD-specific infections are linked to the device itself, including pump, pocket, or percutaneous driveline infection [29]. VAD-related infections include infective endocarditis, mediastinitis, and others occurring in the presence of a mechanical support device [29]. Non-VAD infections are general infections unrelated to the device or heart, which include a broader spectrum such as urinary tract infections, pneumonia, and gastrointestinal infections, among others [29].

In a study performed on 212 patients that underwent LVAD surgery, 48.1% of patients experienced at least one infection post-implantation, with a total of 151 independent infectious events [29]. Among these 151 infections, “49 (32.5%) were VAD-specific, 14 (9.3%) VAD-related, and 89 (58.9%) non-VAD” [29]. Infections encompassed a variety of organisms, including 169 bacteria, 6 fungi, and 3 viruses [29]. Among bacterial infections, 33.7% were Staphylococcus, which was the most prevalent organism, of which seven were methicillin resistant (12.3%) [29]. Other bacteria reported were Pseudomonas (10.7%), Streptococcus (9.5%), Klebsiella (8.3%), and Enterococcus (6.5%). There were also six fungal infections, five of which were Candida, and one was Aspergillus. Nevertheless, three viruses were identified, one influenza A, one respiratory syncytial virus (RSV), and one unknown virus type [29]. According to Wadiwala et al. (2024), older age, diabetes, larger body mass index, renal failure, malnutrition, and prolonged duration of LVAD support have been associated with an increased risk of driveline infection [30].

For management of infections, the first-line therapy is systemic antibiotics, which have a 27% success rate [30]. However, for those unresponsive to antibiotics, a combination of IV antibiotics, surgical debridement, driveline repositioning, utilization of negative pressure wound therapy, muscle flap coverage, or in cases with extension to the pump pocket, driveline or pump exchange is provided [30]. A new approach to treat infections post-LVAD implantation has been studied and consists of “calcium sulfate-based (CS) absorbable beads (Stimulan^®^ Rapid cure: Biocomposites Inc., Wilmington, NC, USA) impregnated with Vancomycin and Tobramycin” [30]. In this study, five patients with LVAD driveline infections refractory to systemic antibiotics were treated with surgical debridement, driveline relocation, and usage of absorbable antibiotic beads [30]. These beads do not need to be removed or exchanged, thus allowing primary closure of the wound [30]. Four of these patients had complete resolution of infection and one of them had a recurrent infection at 7 months at a different part of the driveline that required repeat debridement and placement of bead [30].

Beads are of two types: “non-absorbable beads made of polymethyl methacrylate (PMMA) and absorbable beads made of calcium sulfate (Stimulan^®^ Rapid cure)” [30]. Both types of beads effectively provide a high concentration of antibiotics locally; however, there are several differences between them [30]. First, while using PMMA beads, cautious selection of the antibiotics is required, as antibiotics used with PMMA must be heat-stable due to the exothermic polymerization reaction, water-soluble for dissolution out of the PMMA and available in powder form for admixing them into the polymer powder [30]. Antibiotics often used in PMMA are broad spectrum, including gentamycin, Vancomycin, and Tobramycin [30].

f. Arrhythmias:

According to Ben Gal et al. (2021), arrhythmias are common in LVAD patients, and 30–40% of patients are hospitalized within the first two years after implantation. Atrial fibrillation (AF) is manifested atypically in LVAD-supported patients, and anticoagulation is rarely an issue [23]. In rare cases, if RV function is marginal, AF may become acutely symptomatic, and may lead to exercise intolerance [23]. AF in LVAD patients is treated using the same principles as in non-LVAD patients, except that ablation is rarely considered [23]. A total of 20–50% of LVAD recipients developed ventricular tachycardia or fibrillation (VT/VF) with ventricular arrhythmias prior to the implant being the main predisposing factor [23]. VT/VF may also arise because of underlying heart disease, scar tissue related to the LVAD operation, or mechanical irritation of the LV inflow cannula [23]. Management of LVAD patients with VT/VF is dependent on the patient’s clinical presentation. We first need to ensure electrocardiogram monitoring and IV access, and place a magnet over the implantable cardioverter defibrillator (ICD) if the patient has an ICD to prevent discharges [23]. If the patient is hemodynamically stable, IV amiodarone and reduction in pump speed is considered [23]. However, if not responding, we should perform sedation and cardioversion, concurrently with correction of acidosis, hypokalemia, and hypomagnesaemia [23].

In a retrospective cohort study on all patients who underwent LVAD implantation at the Mayo Clinic, 883 patients were included to determine the incidence of electric storm (ES) [31]. “The ES was defined as ≥3 episodes of sustained ventricular tachycardia (VT) or ventricular fibrillation over a 24h period requiring increased dosing of an antiarrhythmic, ICD shock or external defibrillator shock or antitachycardia pacing” [31]. The results of the study showed that ES occurred in 7% (*n* = 61) of patients with a median of 13 days following surgery [31]. A total of 57% of patients (*n* = 35) had ES within 30 days, and 43% of patients (*n* = 26) developed ES at a median of 545 days post-surgery [31]. Among those developing ES, 26% of them died within 1 year [31]. A history of ventricular arrhythmias and ICD shocks before the procedure was significantly associated with developing ES [31]. Recurrent ES after an initial episode of ES occurred in 30% of patients in the study [31].

g. Right-sided heart failure

Late right-sided HF can occur in 15–20% of LVAD patients. Treatment includes increasing dosage of diuretics along with inotropic support if needed [23] Aortic regurgitation that occurs in the setting of LVAD patients can lead to RHF, as the incomplete unloading of the LV leads to pulmonary artery hypertension affecting the RV function. A retrospective single-center study including 336 patients with late-onset right-sided ventricular failure (LORVF) found that diabetes mellitus, a body mass index > 29 and blood urea nitrogen level > 41 mg/dL are significant predictors of LORVF [5].

Therefore, according to EACTS 2019 guidelines, routine follow-up echocardiography for assessment of right heart function has been recommended with class I evidence [5]. Additionally, invasive hemodynamic measurements should be considered. It is recommended to start the initial treatment for right heart failure with diuretics with class I evidence, and urgent listing for a heart transplant is recommended if the patient is a transplant candidate [5] In patients with pulmonary hypertension, drugs that decrease the pulmonary vascular resistance should be administered, including inhaled NO and sildenafil [19]

h. Pseudoaneurysm:

Another complication related to LVAD implantation is formation pseudoaneurysm at the outflow site or ventricular pseudoaneurysm [32]. In 2016, Kajy et al. (2024) presented a case of a man in his thirties who was fitted with a HeartMate-II left ventricular assist device (Thoratec Corporation, Pleasanton, CA, USA) for end-stage non-ischemic dilated cardiomyopathy. Multiple instances of suspected and proven pump thromboses exacerbated his LVAD therapy, necessitating a device swap with a HeartMate-II in 2017 [32]. Due to a recurring pump thrombosis in 2018, the patient had a left thoracotomy pump exchange with a HeartMate-III [32]. A computed tomography scan of the abdomen and thorax showed a hematoma encircling a sizable left ventricular pseudoaneurysm, spanning 10 × 10 × 8 cm in diameter [32]. This patient’s medical management goal was to unload the ventricle and reduce afterload [32]. Therefore, antihypertensive medications were optimized to target a MAP goal of 65 mm Hg in order to lower left ventricular wall stress [32]. Also, LVAD pump speed was increased to decompress and unload the left ventricle, reducing blood flow in the pseudoaneurysm [32].

Kurdy et al. (2024) address a patient who underwent surgical implantation of a HeartMate-II LVAD and closure of the aortic valve by a pledged suture. His EF improved to 45%, so the device was explanted [33]. However, the explantation was complicated by LV wall insufficiency and tearing, requiring repair with an Amplatzer plug [33]. “Compared to true ventricular aneurysms, pseudoaneurysms may demonstrate a narrow neck at their origin from the ventricular wall” [33]. Additional findings included hemopericardium on cross-sectional and dynamic imaging [33]. In order to repair the pseudoaneurysm, the patient underwent percutaneous closure of the defect; two Amplatzer plugs were used due to the size of the pseudoaneurysm and the diameter of the neck [33]. “Four days later, the patient underwent cardiopulmonary bypass and left anterior thoracotomy, removal of the Amplatzer plugs, drainage of a large preperitoneal fluid collection, and closure of the LV apex” [33].

## 12. Clinical Outcomes with LVADs

a. Survival:

Clinical outcomes with left ventricular assist devices (LVADs) have significantly improved for patients with advanced heart failure, particularly with advancements in device technology such as the HeartMate 3. Studies have shown substantial improvements in survival rates, quality of life, and functional status. Namely, the MOMENTUM 3 trial demonstrated that among 366 patients, the HeartMate 3’s 2-year survival free from disabling stroke or reoperation was 77.9% compared to 56.4% for the HeartMate II [34]. With the newer device, pump thrombosis and ischemic stroke were significantly reduced. After this trial, HVAD production was discontinued. Moreover, given a cohort of 89 patients who received an HMII LVAD between February 2004 and December 2010, Hanke et al., 2018 [35] found a survival rate of 71% one year after HeartMate II implantation, 65% at the second year, 63% at the third year, 56% at the fourth year, and 54% after five years of LVAD support. Out of all the patients, 15 remained on device therapy, 39 died, 28 underwent heart transplantation, and 7 had the device explanted for recovery after five years. These results show the durability of LVAD, but also the challenge in following a cohort of patients and accurately determining the long-term clinical outcomes of LVAD. Moreover, the most common adverse events were bleeding (68%) and LVAD infection (49%) in addition to seven cases of pump thrombosis (8%) [35]. The CLEAR-LVAD study, conducted by Pagani et al., retrospectively analyzed Medicare claims and device registration data for patients who received de novo, durable LVAD implants between January 2014 and December 2018. Among the 4195 patients studied, those with the HeartMate 3 LVAD had a significantly lower adjusted hazard ratio for mortality at 1 year compared to HeartMate II (HR: 0.64; 95% CI: 0.52–0.79) and other LVADs (HR: 0.51; 95% CI: 0.42–0.63). Additionally, the HeartMate 3 group experienced fewer hospitalizations and hospital days per patient-year, leading to a reduction in Medicare expenditures by 17.4% compared to HeartMate II and 26.1% compared to other LVADs. Therefore, in accordance with the above data, HeartMate 3 LVAD is associated with superior survival, reduced healthcare resource use, and lower healthcare expenditures compared to other LVADs [36].

A comprehensive analysis of the clinical outcomes associated with a fully magnetically levitated LVAD compared to an axial-flow LVAD in patients with advanced heart failure shows a significant improvement in patient outcomes with the magnetically levitated LVAD. Specifically, the per-protocol analysis revealed that the composite outcome of survival to transplant, recovery, or LVAD support free of debilitating stroke or reoperation to replace the pump occurred in 54.0% of patients with the magnetically levitated LVAD, compared to 29.7% with the axial-flow LVAD (hazard ratio, 0.55). Additionally, overall survival was 58.4% in the magnetically levitated group versus 43.7% in the axial-flow group (hazard ratio, 0.72). These results were statistically significant, indicating a better composite outcome and higher likelihood of overall survival at 5 years with the magnetically levitated centrifugal-flow LVAD [37]. Another study analyzed the effects of neurohormonal blockade (NHB) on patients with heart failure who are supported by LVAD, focusing on patients with continuous flow LVADs from 2008 to 2016. It revealed that NHB use is associated with a significantly lower risk of death and improved quality of life. Patients receiving combination therapy with an ACEi or ARB, β-blocker, and mineralocorticoid antagonist showed the highest survival at 4 years. The study concludes that both NHB and LVAD support offer a potential synergy for enhancing patient outcomes in advanced heart failure [38].

b. Quality of life:

Recent studies on LVADs have explored their profound impact on patient quality of life (QoL) across multiple dimensions. These devices have become an alternative for patients with advanced heart failure, particularly as destination therapy (DT) for those ineligible for transplants [39]. By offering enhanced survival rates and better hemocompatibility profiles due to technological advancements like smaller device sizes and improved durability, these devices improved QoL and prognosis [10]. Patients undergo significant psychological and social adjustments, facing distinct challenges during different stages: pre-LVAD, implantation hospitalization, early home adaptation, and late home adaptation; despite the emotional distress associated with each stage, patients often adapt by effective coping strategies and social support [40]. Furthermore, the review by Adams and Wrightson (2018) [41] points out that the assessment of QoL should include both patient and caregiver perspectives to correctly study the impact of LVADs, and this is supported by recent studies that call for comprehensive views in order to understand and enhance patient care [39].

In an extensive study by [42] on the QoL, patients with LVADs reported compromised health-related quality of life (HR-QoL) and sexual dysfunction, regardless of how long they had been using the device. These issues often persisted and even slightly worsened over time. Despite this, patients experienced fewer heart failure (HF) symptoms and improved their capacity to perform daily tasks and engage in social activities. Differences in baseline characteristics and comorbidities among various patient cohorts might explain the inconsistencies in questionnaire data. Nonetheless, survey scores showed relative improvement compared to pre-LVAD implantation HR-QoL data. For example, patients with HF and reduced ejection fraction in New York Heart Association Class IV had a mean Kansas City Cardiomyopathy Questionnaire (KCCQ) summary score of 29 [43], which is considerably lower than scores observed in LVAD-supported patients. Enhancing intimacy and sexual activity can significantly improve quality of life for patients and their partners. Providing pre-LVAD implantation education and consultation on future intimate relationships could address concerns and potentially enhance HR-QoL. LVAD-supported patients tend to engage more frequently in social activities with family and friends compared to the pre-implantation period, with many leading active social lives. There has also been an increase in domestic travel among these patients, likely reflecting their increased independence and sense of security [42].

c. Functional status:

A study on continuous flow LVADs shows that most patients experience significant improvements in their NYHA class, moving from class IV to classes I or II within six months. These benefits extend to exercise capacity and overall quality of life over a 24-month period [44]. Similarly, a review on exercise and physical therapy indicates that tailored exercise programs greatly enhance cardiopulmonary exercise capacity and functional independence in LVAD patients. These programs are proven to improve physical performance and daily living activities, crucial for long-term health [45]. Additionally, a clinical trial protocol for functional training in heart failure patients, including those with LVADs, aims to measure gains in VO₂ max, muscle strength, and independence through a structured regimen, showing promise for rehabilitation [46]. Furthermore, an expert panel on cardiac rehabilitation recommends comprehensive rehab programs that address both physical and psychological needs for heart failure patients with LVADs. Such programs are said to enhance exercise tolerance and independence, vital for patient recovery and quality of life [47]. Lastly, a study on intravenous iron therapy in heart failure patients, including those with LVADs, reports significant improvements in NYHA class, exercise endurance, and daily functioning, suggesting that adjunct therapies can further boost rehabilitation outcomes [48].

## 13. Technological and Biomedical Advances

Recent technological and biomedical advances in LVADs have significantly enhanced their design, functionality, and overall patient outcomes. These advancements include strides in miniaturization, increased durability, reduced invasiveness, and prospects for fully implantable systems. One notable innovation in LVAD design is miniaturization. Modern LVADs have become significantly smaller and lighter, allowing for implantation in a broader range of patients, including those with smaller body sizes who were previously ineligible. The HeartMate 3 LVAD, for instance, is a compact device that has shown excellent outcomes in terms of survival and quality of life improvements [49]. This reduction in size not only makes the implantation procedure less invasive but also decreases the risk of infection and other complications associated with larger devices.

Increased durability of LVADs is another critical advancement. Innovations in materials science and engineering have led to the development of more robust devices that can operate efficiently over extended periods. The use of magnetic levitation technology in devices like the HeartMate 3 reduces wear and tear, leading to longer device lifespan and fewer mechanical failures [50]. This improvement translates to fewer surgical interventions for patients following initial insertion and more stable management of heart failure.

Reduced invasiveness has also been a significant focus in recent LVAD advancements. Techniques such as minimally invasive surgery have become more prevalent, reducing recovery times and surgical risks. For example, the lateral thoracotomy approach, which avoids a full sternotomy, has been increasingly adopted, resulting in less postoperative pain and quicker recovery [16].

Regarding future prospects, LVAD technology appears promising, with several potential advancements on the horizon. One is the development of fully implantable LVAD systems, unlike the current LVADs that require an external power source and driveline. Fully implantable systems are in development and are certain to improve patient quality of life [51]. Wireless energy transfer technology is a key area of research in this context, aiming to power the device through transcutaneous energy transmission (TET) systems. A notable example is FineHeart’s ICOMS technology, which has successfully demonstrated the ability to power a cardiac assist device via a TET system. It is minimally invasive, has low energy consumption, and does not cause tissue heating. Hence, it is a promising solution for fragile patients. The TET system was tested for seven days, with results showing minimal temperature increases that posed no risk of tissue damage or infection, thereby validating its safety and efficacy [51]. Similarly, Abbott and Resonant Link’s collaboration on a Fully Implantable Left-Ventricular Assist System (FILVAS) represents another leap forward. Their system uses Resonant Link’s high-efficiency wireless power transfer technology to power the LVAD and recharge its internal battery, meeting strict thermal and operational standards. This technology not only allows continuous operation even with body movement but also ensures that the device’s temperature remains within safe limits. The FILVAS aims to provide LVAD patients with a higher quality of life by eliminating external components and offering reliable, untethered power [52].

Another promising advancement is enhancing LVADs with artificial intelligence (AI) and machine learning (ML) to optimize performance by continuously monitoring the patient’s physiological parameters and adjusting the device settings in real time to match the demand. For example, AI algorithms could predict adverse events or device malfunctions before they occur, allowing for proactive management and improved outcomes [53].

Further prospects include advancements in biomaterials used in LVAD construction. Research into biocompatible and bioresorbable materials aims to create devices that can better integrate with the body, reducing the risk of rejection and other immune responses. Additionally, improvements in battery technology are expected to extend the duration between recharges, enhancing the convenience and mobility for patients [50].

Gene therapy is another frontier being explored in conjunction with LVAD technology. The idea is to use gene-editing tools like CRISPR to correct underlying genetic causes of heart failure, potentially reducing or even eliminating the need for LVADs in some patients. While still in the experimental stages, such therapies hold the promise of more permanent solutions to heart disease [54].

The integration of LVADs with personalized treatment plans for the management of heart failure could optimize and potentially improve patient survival and quality of life. For instance, integrating LVAD therapy with personalized drug regimens can enhance heart function and reduce complications. The use of advanced imaging and diagnostic tools to monitor heart function and tailor LVAD settings to the patient’s specific needs is another promising approach. This level of customization ensures that each patient receives the most effective treatment based on their unique physiological and genetic characteristics [55]. Patients can receive personalized treatment with warfarin and/or aspirin dosage by reference to their genotypes in polymorphisms of VKORC1 and UGT1A6 genes [56]. This demonstrates the potential to use genetic analysis and machine learning to personalize patient care.

## 14. Ethical and Economic Considerations

a. Ethical Issues

LVADs have become a vital technology in treating advanced heart failure, but they also present numerous ethical challenges, especially regarding end-of-life care and decision making. One major ethical concern is patient autonomy and informed consent. The complexity of LVAD technology and its potential complications require thorough patient education to ensure truly informed consent [57]. Patients need to understand not only the benefits but also the risks, such as infection, bleeding, and stroke, that come with LVAD implantation.

Another significant ethical issue is the allocation of resources. The high costs and limited availability of LVADs make deciding who receives these devices a moral challenge. This is especially critical in cases where the potential benefit is uncertain, requiring a balance between the chance of extending life and the quality of that extended life [58]. Furthermore, the decision to deactivate an LVAD at the end of life raises profound ethical questions. Such decisions often involve determining when continued intervention becomes futile, balancing the prolongation of life with the need to ensure a dignified death [59].

Healthcare professionals also face ethical dilemmas when managing LVAD complications. The principle of non-maleficence, or “do no harm,” must constantly be weighed against the potential benefits of aggressive interventions needed to manage LVAD-related issues. This can create tension between keeping a patient alive and possibly causing further suffering and reduced quality of life [60].

b. Cost-Effectiveness

The economic impact of LVADs on healthcare systems is considerable, significantly affecting long-term care costs and resource allocation. The process of LVAD implantation and the subsequent care involves substantial initial and ongoing expenses, which include surgery, hospitalization, device maintenance, and the management of complications [60]. On average, the cost of LVAD implantation is around USD 175,420. While LVADs have been shown to increase quality-adjusted life-years (QALYs), they also lead to significantly higher lifetime costs due to frequent readmissions and the need for costly follow-up care. The incremental cost-effectiveness ratio (ICER) is USD 209,400 per QALY gained and USD 597,400 per life-year gained [61]. Similarly, Oz et al. (2003) found that hospital non-survivors incurred approximately double the costs (USD 315,015) compared to hospital survivors (USD 159,271) [62].

Long-term care costs are a critical factor in evaluating the economic impact of LVADs. Patients with LVADs often need frequent medical monitoring and management of complications, leading to recurring healthcare expenses. However, studies suggest that these costs may be offset by reductions in hospital readmissions and emergency care for advanced heart failure symptoms [63]. Additionally, advancements in LVAD technology and care protocols are continually improving device durability and patient outcomes, potentially enhancing cost-effectiveness over time. For bridge-to-transplantation (BTT) patients, estimated incremental cost-effectiveness ratio (ICER) reductions have exceeded 50% and are approaching the USD 50,000/QALY threshold [64]

## 15. Conclusions

In summary, LVADs have played a major role in improving survival and quality of life in people with advanced heart failure, refractory to medications. Different types and generations of LVAD have been created with time, pulsatile and continuous flow, each of which has its advantages and disadvantages. Traditionally, implantation occurred through median sternotomy; however, less invasive techniques such as percutaneous implantation and sternum sparring techniques have been brought up for application. Several complications can occur post-implantation such as infection, pump thrombosis, bleeding and others, making good pre- and post-operative care a necessity. Candidates for LVAD implantation should be selected carefully by a multi-disciplinary and qualified team according to guidelines, to maximize beneficence and reduce complications. The main question remains about how to reduce the risk of post-operative complications of LVAD implantation. Is it by creating other generations of devices? Or can artificial intelligence have a promising role in the field? Could it help in the early detection of complications to assure timely management?

## Figures and Tables

**Table 4:** Differences between intra-pump and outflow graft thrombosis [22].

	Intra Pump Thrombosis	Outflow Graft Thrombosis
Signs	Progressive exertional dyspnea	Signs of distal embolization
Duration	Short	Long
Hemolysis	Increased LDH, Low Hemoglobin, increased plasma free hemoglobin, dark urine	Absent
Echocardiographic findings	Inflow cannula turbulence with visible thrombus	Outflow cannula turbulence and no visible thrombus inside the LV
CT angiography	No outflow graft obstruction	Outflow graft stenosis, obstruction
LVAD parameters	Low flow alarm and decrease in power spikes	

## Data Availability

Not applicable.
